# Engaging Patients and Caregivers Managing Rare Diseases to Improve the Methods of Clinical Guideline Development: A Research Protocol

**DOI:** 10.2196/resprot.6902

**Published:** 2017-04-28

**Authors:** Dmitry Khodyakov, Kathi Kinnett, Sean Grant, Ann Lucas, Ann Martin, Brian Denger, Holly Peay, Ian Coulter, Arlene Fink

**Affiliations:** ^1^ RAND Health Santa Monica, CA United States; ^2^ Parent Project Muscular Dystrophy Hackensack, NJ United States; ^3^ RTI International Research Triangle Park, NC United States; ^4^ RAND Santa Monica, CA United States; ^5^ University of California Los Angeles Los Angeles, CA United States

**Keywords:** Delphi method, Duchenne muscular dystrophy, ExpertLens, guideline development, online stakeholder engagement panels, patient engagement

## Abstract

**Background:**

Clinical guidelines provide systematically developed recommendations for deciding on appropriate health care options for specific conditions and clinical circumstances. Up until recently, patients and caregivers have rarely been included in the process of developing care guidelines.

**Objective:**

This project will develop and test a new online method for including patients and their caregivers in this process using Duchenne muscular dystrophy (DMD) care guidelines as an example. The new method will mirror and complement the RAND/UCLA Appropriateness Method (RAM)—the gold standard approach for conducting clinical expert panels that uses a modified Delphi format. RAM is often used in clinical guideline development to determine care appropriateness and necessity in situations where existing clinical evidence is uncertain, weak, or unavailable.

**Methods:**

To develop the new method for engaging patients and their caregivers in guideline development, we will first conduct interviews with experts on RAM, guideline development, patient engagement, and patient-centeredness and engage with Duchenne patients and caregivers to identify how RAM should be modified for the purposes of patient engagement and what rating criteria should patients and caregivers use to provide their input during the process of guideline development. Once the new method is piloted, we will test it by conducting two concurrently run patient/caregiver panels that will rate patient-centeredness of a subset of DMD care management recommendations already deemed clinically appropriate and necessary. The ExpertLens™ system—a previously evaluated online modified Delphi system that combines two rounds of rating with a round of feedback and moderated online discussions—will be used to conduct these panels. In addition to developing and testing the new engagement method, we will work with the members of our project’s Advisory Board to generate a list of best practices for enhancing the level of patient and caregiver involvement in the guideline development process. We will solicit input on these best practice from Duchenne patients, caregivers, and clinicians by conducting a series of round-table discussions and making a presentation at an annual conference on Duchenne.

**Results:**

The study protocol was reviewed by RAND’s Human Subjects Protection Committee, which determined it to be exempt from review. Interviews with RAM experts have been completed. The projected study completion date is May 2020.

**Conclusions:**

We expect that the new method will make it easier to engage large numbers of patients and caregivers in the process of guideline development in a rigorous and culturally appropriate manner that is consistent with the way clinicians participate in guideline development. Moreover, this project will develop best practices that could help involve patients and caregivers in the clinical guideline development process in other clinical areas, thereby facilitating the work of guideline developers.

## Introduction

Clinical guidelines provide systematically developed recommendations for deciding on appropriate health care options for specific conditions and clinical circumstances [1]. A key methodological aspect that influences the quality of guideline recommendations is the composition of the group developing the guideline [2]. All stakeholders with a legitimate interest in a clinical guideline should be engaged in guideline development to ensure that guidelines are created in a transparent, democratic manner and are acceptable to different stakeholder groups [3]. However, clinical guideline development groups traditionally have not directly involved patients or their caregivers [4]. For example, only an estimated 25% of guidelines involve patients in the development process [4]. Moreover, a review of 51 evidence-based clinical practice guidelines found only 5% of guideline word count and 6% of references were related to patient preferences [5].

Patients, their caregivers, and many organizations concerned about guideline development have long argued that guideline development groups need to better include patients and caregivers because these stakeholders have particular knowledge and expertise on the direct experience of conditions of interest [1]. Research shows that patients and clinicians value the balance between risks and benefits differently [6] and that patients and their families provide unique perspectives that may differ from areas of focus in a clinical encounter [7]. For instance, while some care recommendations might be deemed appropriate and necessary by clinicians, they may not be acceptable from the patient perspective [8]; patients may focus more on issues related to overall quality of life rather than specific disease areas or life expectancy [9]. Effective implementation of guidelines ultimately requires patient adherence, and one might argue that the practical use of guidelines will be higher where patients feel the guidelines are sensitive and relevant to their needs. Moreover, the World Health Organization, National Institute for Health and Care Excellence (NICE), and Institute of Medicine also called for involving patients and other public stakeholders in developing and implementing clinical guidelines [4,8,10]. For example, the Guideline International Network (G-I-N)—an international organization dedicated to guidelines and to hosting the largest international guideline library—created a Patient and Public Involvement Group (G-I-N PUBLIC) to more effectively engage patient stakeholders in developing and implementing clinical guidelines [11]. Finally, the Grading of Recommendations Assessment, Development, and Evaluation (GRADE) approach to guideline development also encourages guideline developers to ensure that guidelines address the outcomes that patients value and that their recommendations are likely to be acceptable from the patient perspective [12].

While there is agreement that patients should be involved in guideline development, there is no consensus on how patients should participate in this process. Patients can be involved at different stages of the process, from topic selection, to reviewing and grading the strength of evidence, to developing recommendations [13]. They can also be asked to provide their views on living with their condition, accessing services, perceived benefits and harms of treatment options, or clinical outcomes of importance [1,14]. Perhaps more than any other stakeholder, patients are able to reflect on what outcomes they are looking for from the guidelines. Besides asking patients to join the evidence review group [15] or to submit evidence to be considered for guideline development [1], which could lead to a broader range of evidence being considered, guideline development groups have engaged patients in reviewing existing studies on patient preferences and solicited patient input in designing data collection instruments to help identify areas where patients and their caregivers feel guideline recommendations are most needed [16]. Some guideline groups have dedicated time during meetings to focus on patient and caregiver perspectives [17]. For example, NICE uses deliberative participation methods that involve members of the general public, including patients, in discussing social values related to clinical guideline development, so panel experts can interact directly with citizens [18]. Some guideline organizations include professional advocates acting on behalf of patients with a given condition in guideline development groups or panels to promote “the patient perspective” as an influence on guideline recommendations [19]. Although professional patient advocates play an important role, lay people with a given medical condition should also be directly engaged in the process of developing guideline recommendations [20] because when they are excluded, trade-offs on what makes the cut as a recommendation are made on behalf of patients rather than with and by patients [21]. Finally, it is generally not possible for one person or a small number of people to adequately represent the diversity of perspectives of all patients with the condition. Therefore, approaches that encourage participation of larger groups of patients are needed.

In summary, research is needed to develop a systematic, scalable, and culturally appropriate method to engage patients—particularly those with rare diseases or disabilities that limit their mobility—and their caregivers in developing guideline recommendations. Ideally, this method should facilitate the practical use of care guidelines given low levels of compliance and adherence to care recommendations among both clinicians and patients [22,23]. Finally, this new method should be consistent with the method used by clinicians in the process of developing consensus-based clinical guidelines and account for key recommendations from a workshop of international leaders in guideline development that recommended to expand patient engagement methods to include Web-based consultations and to analyze the benefits and drawbacks of specific methods for patient involvement [13].

In this project, we will develop such a method using Duchenne muscular dystrophy (DMD) as an example. DMD is a progressive, fatal disorder where caregiving, financial, emotional, and physical demands increase over time and can impact the entire family. Affected individuals have progressive loss of functional muscle fibers, which results in weakness, loss of ambulation (typically in the teen years), and premature death (typically in the mid-to-late second decade of life) [24]. The DMD community developed a set of clinical care guidelines covering 8 domains of care [25,26], but patients and caregivers have been consulted in the development of guidelines for 2 domains only.

## Methods

### Overview

This project is an equal partnership between researchers from RAND, a nonprofit research institution that developed Delphi and RAND/UCLA Appropriateness Method (RAM), and community partners from Parent Project Muscular Dystrophy (PPMD), the largest most comprehensive nonprofit organization in the United States focused on finding a cure for DMD. The partnership is a natural fit given the complementary expertise, skills, and resources both organizations bring to the partnership. We assembled a strong interdisciplinary team of academic and community investigators, along with patient and caregiver representatives, who bring the right mix of methodological skills and clinical expertise combined with the lived experience of caring for a Duchenne patient. The project has a 7-person interdisciplinary Advisory Board that includes an adult DMD patient, caregivers and patient advocates, researchers, a clinician, a guideline developer, and a RAM expert.

### Data Collection

Our study relies on a 3-step mixed-methods approach to develop and test a new approach for patient engagement in clinical guideline development.

First, we will adapt RAM, a gold standard approach used by clinical experts in the process of guideline development to reach consensus on appropriateness and necessity of care recommendations [27], for the purposes of systematic online engagement of DMD patients and their caregivers in the process of determining patient-centeredness of already existing care guidelines. RAM is a modified Delphi method that combines two rating rounds with a face-to-face moderated discussion. Nine clinical experts review the existing evidence (if any) and rate the appropriateness and sometimes necessity of existing treatment options or procedures using a 9-point Likert scale. Appropriateness and necessity ratings are based on experts’ own clinical judgments—informed by the systematic review of existing evidence—about what treatment options are best for “an average patient presenting to an average physician who performs the procedure in an average hospital” [27]. RAM is considered a formal consensus exploration method that meets the requirements of a scientific method; it has been recommended for use in guideline development in the absence of rigorously conducted randomized controlled trials [28] because it helps provide explicit links between the scientific evidence and the guideline recommendation. RAM was used to develop Duchenne guidelines. RAM panels, however, have been criticized for their small size and inclusion of only clinicians and researchers [29].

In modifying RAM, we will consult with up to 10 researchers and clinicians who have used RAM in the past, engaged patients in guideline development, or worked on topics related to patient-centeredness. We will solicit their perspectives on what modifications to RAM are needed to facilitate patient and caregiver engagement in guideline development, how patient-centeredness ratings of care management strategies can be included in guidelines, how guidelines can be rated and perceived by the clinical community, and how these ratings could help providers, patients, and their caregivers determine the best course of action and ensure adherence to care recommendations.

To triangulate these findings, we will also solicit input from a maximum variation purposive sample of up to 10 adult DMD patients and up to 30 caregivers. We can achieve diversity of perspectives and experiences by recruiting adult patients and caregivers of patients at various stages of disease progression, which is typically associated with the patient’s age, and from different geographic locations. Participants will be recruited by PPMD through its Duchenne Connect (DCN) registry—the largest repository of patient self-reported information on DMD.

We will be asking patients and caregivers to share their perspectives on the topics covered during the RAM expert interviews and comment on the usability of and suggest modifications to the ExpertLens (EL) system, an online modified Delphi platform that we will use for patient and caregiver engagement. EL is a previously evaluated online modified Delphi system that typically combines two rounds of rating with a round of asynchronous moderated online discussions [30,31]. EL has been used in numerous research studies [32-39] but has yet to be used in the context of patient involvement in clinical guideline development. We chose EL because it allows for conducting RAM panels and soliciting input from large, diverse, and geographically distributed groups of participants iteratively; for combining quantitative and qualitative data; for engaging participants anonymously; and for exploring points of agreement and disagreement among participants [40,41]. These characteristics make EL particularly useful for engaging DMD patients and caregivers—who are located around the country, with some living abroad—and for collecting their input on existing care guidelines for DMD. Patients and caregivers will rate patient-centeredness of care recommendations using 9-point Likert scales and share their thoughts using online discussion boards that use the same open-ended format as previous PPMD engagement efforts.

To learn what DMD patients and their caregivers think about EL, we will first ask them to watch a short video describing each EL round and what participation in the panel will entail. We will then provide them with access to the EL system and ask them to share their thoughts on the user-friendliness of the EL tool, the instructions on how to use EL, the statistical feedback that will be provided to participants, and the interactiveness of the discussion round, among other topics. To do this, participants will answer a series of open-ended questions and join threaded discussion boards within EL. We will also ask questions about participants’ understanding of and thoughts about patient-centeredness, participation burden in the EL process that is likely to be acceptable from the perspective of patients and caregivers, the maximum number of clinical scenarios, and the amount and type of background information on DMD patient and caregiver preferences and clinical information that should be included. We anticipate that participants will spend approximately 1 to 2 hours answering these questions and engaging in an online discussion over a period of 7 to 10 days. They will receive a $50 gift card for their participation.

Based on the input obtained from expert interviews and DMD patients and caregivers, we will implement changes to the EL platform and develop the modified Delphi protocol for rating patient-centeredness of already developed clinical guidelines. The revised version of the EL will be pilot tested by 2 to 3 DMD patients and 7 to 8 caregivers, who will go through all three rounds of the EL process as if they were real study participants. After each round, pilot-testers will share feedback by answering questions either by email or phone. These open-ended questions will focus on specific issues related to system usability, question clarity, and ease of discussion use, among other topics. We anticipate that participation in all three rounds will take about 3 to 4 hours over a period of 3 weeks. Participants will receive a $50 gift card for each round completed at the end of the pilot.

Second, we will test the new approach using one of the DMD care guideline domains that was developed using RAM but without patient or caregiver input, such as cardiac or endocrine care management guidelines. To do so, we will conduct two 3-round EL panels using a modified version of the EL system to determine the level of patient-centeredness of selected DMD care recommendations already deemed clinically appropriate. Our operational definition of patient-centeredness will be informed by the existing literature and consistent with the operationalization of appropriateness used in RAM. Our 3-round design is consistent with a recommendation for conducting Delphi studies with 2 or 3 rating rounds [42]. In testing the new approach, we will compare participants’ ratings of patient-centeredness, satisfaction levels, and participation rates after rounds 1 and 3 in both panels. Because round 1 of the EL process is essentially a survey, we will treat round 1 ratings as data from a patient engagement survey—a more common mode of patient engagement than the modified Delphi approach [43]. Such a strategy is particularly relevant for comparing the two approaches in a rare disease community where the pool of potential participants is limited. Finally, as in previous studies validating the EL approach [31,39], we will determine the replicability of final panel ratings of patient-centeredness by comparing results of two EL panels conducted using the same protocol.

We will use the DCN patient registry and PPMD social media channels to recruit 20 to 25 adult patients and 60 to 70 caregivers with a range of experiences with Duchenne care and varying degrees of comfort using technology. We will then randomly assign them to two panels similar in their composition. Doing so will help us determine the replicability of panel determinations and adhere to the best practice of conducting online modified Delphi panels that suggests including 20 to 40 participants [31] to ensure their active participation while minimizing burden associated with reading comments posted by all panel members. We will recruit more than 40 participants per panel to account for attrition typical for multiround Delphi studies without face-to-face meetings. Our panels will be significantly larger than a traditional 9-person RAM clinical panel [27]. We can engage more participants because of the online nature of the panel, which helps increase the reliability of panel findings [28]. Our goal is to recruit a maximum variation sample [44] that reflects the diversity of DMD patient and caregiver experiences. This sample will not be random, because participants will be chosen on purpose to ensure their knowledge, expertise, and diversity of experiences. This is a standard approach to recruiting Delphi participants [28,45].

**Figure 1:**
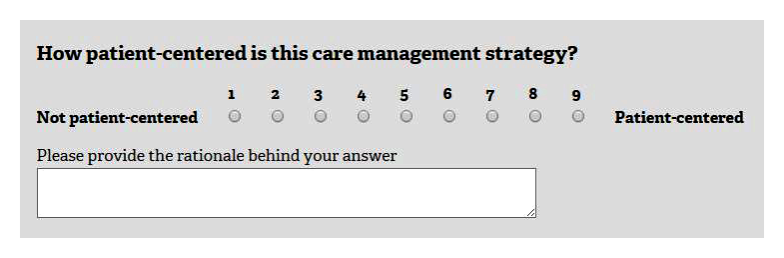
Round 1 mock-up screenshot.

**Figure 2:**
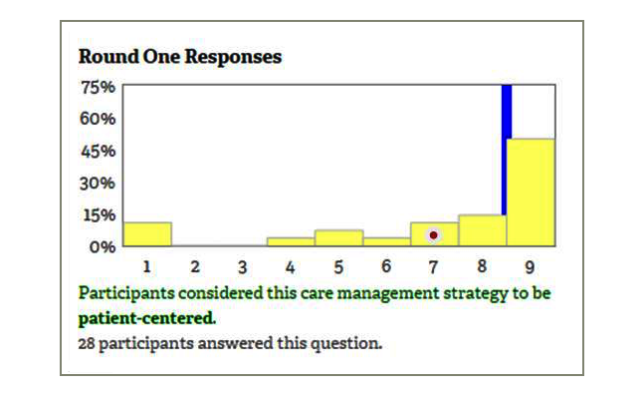
Round 2 mock-up screenshot.

In round 1, participants will use 9-point Likert scales to rate the level of patient-centeredness of selected care management strategies and explain their responses using open-text boxes provided after each rating question (Figure 1). In round 2, participants will see a distribution of responses to all round 1 questions (Figure 2). While only the panelist knows his or her individual rating, all participants know the group’s ratings. Showing statistical feedback to the participant is an essential component of the Delphi process [42]. For each question, participants will see a bar chart showing the frequency of each response category (yellow bars), a group median (blue line), their individual response (red dot), and a short statement describing the group decision based on the group agreement as produced by the RAM [27]. Consistent with best practices in Delphi studies [45], we will provide instructions on how to interpret statistical results using instructional videos and text boxes that appear when a participant hovers over a chart. Participants will be also able to review all rationale comments posted in round 1 and discuss group ratings using an asynchronous and anonymous discussion board moderated by content experts from PPMD/DCN and online engagement experts from RAND. In round 3, participants will reanswer round 1 questions and rate any new care management strategies that might have been suggested in round 2. Allowing for new questions to be added in round 3 is consistent with the best practices for conducting Delphi studies [42]. All participants will receive a $50 gift card for completing each round.

At the end of each EL panel, participants will use 7-point Likert-type scales to rate their satisfaction with the online engagement process [31] by expressing their level of agreement with such statements as “participation in this study was interesting,” “the discussions brought out views I hadn’t considered,” and “I was comfortable expressing my views in the discussion round,” among others. We will use a modified version of these questions after round 1 and 3 to compare participants’ experiences. Modifications to satisfaction questions that are asked after round 1 are needed because participants would not have participated in the discussion round at that time. We will develop additional questions about the usefulness and feasibility of widespread use of the online process of rating patient-centeredness of care guidelines. Open-ended questions will be added to encourage participants to use their own words to share their experiences and perspectives. A subsample of patients and caregivers involved in the EL process will be asked to participate in semistructured phone interviews to further share their experiences and thoughts after they complete all study rounds.

Third, we will develop a series of best practices for engaging patients and their caregivers in the process of care guideline development. To do so, we will identify generalizable lessons learned that could inform the methodology of engaging patients and caregivers in the process of guideline development by working in close collaboration with our study Advisory Board. We will share these best practices during one of the PPMD’s annual Connect Conferences that are attended by nearly 500 families from around the world. During the conference, we will engage with adult DMD patients, caregivers, and clinicians in a series of up to three round-table discussions that will allow us to discuss how to address care management approaches deemed appropriate but not consistent with patient’s care preferences or desired outcomes (eg, not patient-centered). Such small group discussions are a core component of the community-partnered research conference model that we developed for ensuring appropriate dissemination of study findings and for soliciting community input on study outcomes [46]. Each round-table discussion will last for about 60 to 90 minutes and include up to 8 participants. As a token of appreciation, participants will receive a $50 gift card.

### Data Analysis

There are two types of data analysis that will be performed in this study. First, qualitative data from expert and EL participant interviews; feedback from pilot testers about EL; responses to all open-ended questions, rationale comments, and discussions within EL; and round-table discussions will be analyzed thematically by identifying and describing explicit and implicit ideas in the data. Applied thematic analysis [47] will be used because it helps reduce large amounts of textual data and present them in easy-to-understand statements that could be used to explain how experts feel about patient engagement in guideline development, how the EL system should be modified to facilitate patient engagement in care guideline development, how patient input should be solicited, and what participants think about an online approach to patient and caregiver engagement in guideline development once they participate in the study. To expedite the data analysis and ensure its usefulness, we will begin with a deductive approach to directly answer our research questions. At the same time, we will use an open coding strategy to flag interesting ideas and themes that may not be directly relevant to research questions but should be explored further once preliminary data analysis is complete or once more data have been analyzed [48]. Such an inductive approach is crucial for identifying unanticipated ideas and issues that frequently emerge from open-ended questions.

Given the volume of data collected from different types of participants, we will ensure efficient data management by using qualitative data analysis (QDA) software such as MAXQDA (Verbi GmbH) to code and retrieve large amount of textual data to help ensure analysis rigor [49]. Doing so will help us organize, evaluate, code, annotate, and interpret qualitative data by creating easy-to-read reports and data visualizations. We will develop, program within the QDA software, and update on an as-needed basis a codebook—a list of codes, often hierarchically organized, accompanied by a description and examples of each code—to facilitate data coding. Data coders will be trained on how to use the codebook, work jointly to code approximately 20 percent of the data, and discuss any discrepancies until consensus is achieved and the codebook is appropriately adjusted.

Second, ratings of patient-centeredness collected during the EL panel process will be analyzed quantitatively to determine the existence of consensus among participants. It is recommended that every Delphi study determine how consensus will be defined among participants before the data collection begins [42]. One of the EL features is its use of the RAM [27] to automatically determine the group decision (eg, whether a particular care management strategy was deemed patient-centered) for each round 1 item, which is displayed in round 2 and is also calculated after round 3. This process, identical to that used in determining appropriateness of different DMD care management strategies, begins with determining the existence of disagreement among participants using the following a priori process. EL automatically (1) calculates the value of interpercentile range (IPR), or the range of responses that fall between the 70th and the 30th percentiles; (2) calculates the value of the interpercentile range adjusted for symmetry (IPRAS), which is a measure of dispersion for asymmetric distributions; and (3) compares the values of IPR and IPRAS to see if there is disagreement. Disagreement is said to exist if IPR>IPRAS [27,50]. Disagreement among participants automatically produces an uncertain decision. If, instead, there is no disagreement among panelists, the value of the median will determine if the group decision is positive, negative, or uncertain. If the median is within the upper tertile of the 9-point response scale (response categories 7-9), then the decision is positive, meaning that a care management approach is considered to be patient-centered. If the median is within the lower tertile of the 9-point response scale (response categories 1-3), then the decision is negative, meaning that a care management approach is considered to be not patient-centered. A median that lies within the middle tertile (response categories 4-6) produces an uncertain decision. We will use this approach to determining consensus on the patient-centeredness of care management strategies in both EL panels using round 1 and round 3 rating data.

To compare survey and modified Delphi results, we will pool the data across two EL panels and compare determinations of patient-centeredness after round 1 (survey) and round 3 (Delphi). Because there is no right or wrong response, it is not possible to determine which approach produces better results or is more valid at the time the data are collected. However, we follow a recommended practice in Delphi studies and focus on how much each method can help patients and their caregivers reach consensus [28]. To do so, we will first calculate the percentage of care management strategies for which panelists reached agreement versus those where agreement was not achieved. We will then focus on strategies where panelists reached agreement and calculate the proportion with positive, negative, and uncertain determinations. Our assumption is that there will be fewer strategies characterized by participant disagreement after round 3, which we treat as an indicator of the benefit of using the modified Delphi approach. Still, we believe the survey approach may have its own benefit. Indeed, we assume that participant attrition rates (eg, the number of nonresponders) will be smaller in round 1 than in round 3. Larger samples may help provide a better description of the diversity of patient experiences, albeit with potentially fewer qualitative details that will crystalize after round 2 discussion.

To determine the replicability of online panel ratings, we will adopt the following a priori analytic approach originally developed by Shekelle and colleagues [51] to analyze reproducibility of in-person panel ratings. We will first examine round 3 determinations of patient-centeredness for each care management strategy and identify the proportion of strategies receiving positive, negative, or uncertain determinations. Then, we will determine the pairwise percentage of agreement between the two panels and use *t* tests to identify any statistically significant differences in panel ratings. Because the distribution of ratings may be nonnormal, we will conduct sensitivity analyses using the Wilcoxon rank sum, a nonparametric method. We will treat a 70% agreement as an indicator of acceptable reproducibility, which is at least as good as that of in in-person panels [51]. Finally, we will calculate kappa coefficients comparing the determinations made by the two panels across items. If the kappa statistic is at least moderate (.41-.60), we will consider the online approach to be a reliable mode of collecting patient-centeredness ratings using the modified RAM approach [52]. This threshold is conservative; previous research shows that the reproducibility of both in-person [51] and online [31] panel findings is rarely better than moderate.

Besides comparing ratings of patient-centeredness, we will also focus on participant experiences after round 1 (survey) and round 3 (Delphi). As in earlier studies [31], we will pool the data across the two EL panels and look at the average response to each satisfaction question asked in round 1 and round 3. We will consider a mean value of 5 (agree slightly) and higher on the 7-point positively worded agreement scale to be an indicator of a generally positive opinion. Depending on sample size, we may conduct exploratory factor analyses to identify constructs that capture participant experiences and opinions about the new online system. To explore differences in participant satisfaction and perceived usefulness of the online approach after rounds 1 and 3, we will use a paired *t* test. We anticipate greater satisfaction and perceived usefulness after round 3 but a higher level of perceived participation burden.

## Results

The study protocol was reviewed by RAND’s Human Subjects Protection Committee, which determined it to be exempt from review. Interviews with RAM experts have been completed. The study team is in the process of analyzing these interviews. The projected study completion date is May 2020.

## Discussion

Our study is expected to make a number of significant methodological contributions to a growing body of approaches to integrate patients and caregivers as active participants in research teams and decision-making bodies. First, by adapting an existing gold standard approach to expert elicitation for the purposes of systematic engagement of patients and caregivers in a culturally appropriate yet scientifically rigorous manner, our study will help address a methodological gap in evidence on consumer involvement and systematic integration of patient preferences in clinical practice guidelines [4,43]. Second, the proposed project augments and complements efforts to update the existing care and management guidelines for DMD, led by the Centers for Disease Control and Prevention’s DMD Care Considerations Working Group. In the proposed project, we will introduce an innovative new step that could be integrated into care guideline development where patients and their caregivers will rate DMD care management strategies already deemed clinically appropriate and necessary on the patient-centeredness criteria that will be developed in close partnership between patients, clinicians, and researchers. Doing so may help mitigate barriers that have led to variability in guideline implementation and increase guideline adherence, which may lead to improved treatment and quality of life for affected patients and families. Third, the proposed project will develop best practices that could help involve patients and caregivers in the clinical guideline development process in other clinical areas, thereby facilitating the work of groups aiming to incorporate patient values and preferences into guideline development, such as G-I-N and GRADE.

## Abbreviations

DCN: Duchenne Connect
DMD: Duchenne muscular dystrophy
EL: ExpertLens
G-I-N: Guideline International Network
G-I-N PUBLIC: Guideline International Network Patient and Public Involvement Group
GRADE: Grading of Recommendations Assessment, Development, and Evaluation
IPR: interpercentile range
IPRAS: interpercentile range adjusted for symmetry
NICE: National Institute for Health and Care Excellence
PPMD: Parent Project Muscular Dystrophy
QDA: qualitative data analysis
RAM: RAND/UCLA Appropriateness Method
UCLA: University of California Los Angeles
